# Hyperthyroidism-associated hypercalcemic crisis: A case report and review of the literature

**DOI:** 10.1097/MD.0000000000006017

**Published:** 2017-01-27

**Authors:** Ke Chen, Yanhong Xie, Liling Zhao, Zhaohui Mo

**Affiliations:** Department of Endocrinology, Third Xiangya Hospital of Central South University, Changsha, Hunan, China.

**Keywords:** hypercalcemia, hypercalcemic crisis, hyperthyroidism

## Abstract

**Rationale:**

Hyperthyroidism is one of the major clinical causes of hypercalcaemia, however, hyperthyroidism-related hypercalcemic crisis is rare, only 1 case have been reported. The potential mechanisms are still not too clear. It may be related that thyroid hormone stimulate bone turnover, elevate serum calcium, increase urinary and fecal calcium excretion.

**Patient concerns:**

A 58-year-old female patient was found to have Graves’ disease, a marked elevated serum calcium level (adjusted serum calcium: 3.74 mmol/L), and reduced parathyroid hormone level.

**Diagnoses:**

She was diagnosed as hyperthyroidism-associated hypercalcemic crisis.

**Interventions:**

Treatment with methimazole to correct the hyperthyroidism and treatment of the patient's hypercalcaemia was achieved by physiological saline, salmon calcitonin and furosemide.

**Outcomes:**

After treatment for hypercalcaemia and hyperthyroidism, her symptoms and serum calcium levels quickly returned to normal.

**Lessons:**

hyperthyroid-associated hypercalcaemia crisis is rare, however, the diagnosis should pay attention to screening for other diseases caused by hypercalcemia. Timely treatment of hypercalcaemia is a critical step for rapidly control of symptoms, and treatment of hyperthyroidism is beneficial to relief the symptoms and maintain the blood calcium level.

## 1 Introduction

Hyperthyroidism is a cluster of symptoms characterized by the excess level of thyroid hormone in the system, leading to a series of symptoms and signs including palpitation, tremor, weight loss, and complications related to the increased metabolic rate. The bone metabolism is thought to be regulated by thyroid hormone as well. It has been reported that hyperthyroidism is associated with mild to moderate hypercalcemia in approximately 20% of total patients.^[1]^ The serum calcium levels are often increased by a mild to moderate range and it rarely exceeds 3.0 mmol/L in hyperthyroidism associated hypercalcemia.^[2,3]^ Hypercalcemia was defined as a calcium level exceeding 3.5 mmol/L and patient often has symptoms including multiple kidney stones, constipation, and muscle weakness.^[4]^ Severe hypercalcemia or hypercalcemic crisis (serum calcium above 3.5 mmol/L) is considered rare.^[5]^ To date, only 3 cases of hyperthyroidism complicated with hypercalcemic crisis have been reported.^[6–8]^ One case was complicated with hyperparathyroidism and the another was with serum calcium of 3.0 mmol/L.^[6,7]^ Endo et al^[8]^ reported a rare case with serum calcium of 4.1 mmol/L in a patient with hyperthyroidism-associated hypercalcemia. The potential relationship between thyroid hormone and serum calcium level remains obscure. It was speculated that thyroid hormone could directly stimulate bone turnover, elevate serum calcium, as well as urinary and fecal calcium excretion.^[2,9]^

In this report, we presented a case of hyperthyroidism associated hypercalcemic crisis and reviewed the effect of thyroid hormone on metabolism of calcium, phosphate, parathyroid hormone (PTH), and 1,25-OH2-D3.

## 2 Case report

A 58-year-female was admitted to local hospital due to anorexia, nausea, vomiting, and malaise for 1 month. She also complained of palpitations, insomnia, diarrhea, and chest tightness; however, she denied any bone pain. She had lost 15 kg of weight in the month before admission. Her past medical history showed hypertension for 5 years and a cerebral infarction 1 year earlier. She took nitrendipine 10 mg 3 times a day to control her hypertension and denied taking any other medications. There was no family history of thyroid disease, hypercalcemia or malignancy. Laboratory findings showed a significantly elevated free T3 of 15.7 pmol/L (FT3, normal range: 3.1–6.8 pmol/L), elevated free T4 of 36.6 pmol/L (FT4, normal range: 10.3–22.6 pmol/L) and reduced thyroid stimulation hormone of 0.011 μIU/mL (TSH, normal range: 0.27–4.20 μIU/mL). Electrolyte tests showed markedly high serum calcium of 2.99 mmol/L (normal range: 2.08–2.80 mmol/L) and normal serum magnesium, sodium, potassium, and phosphate concentrations. Her serum creatinine was 118 μmol/L (normal range: 45–104 μmol/L), urea nitrogen 9.73 mmol/L (normal range: 1.84–7.14 mmol/L), and uric acid 511 μmol/L (normal range: 155–428 μmol/L). The patient's blood, urine, and stool examinations were all normal. The patient was diagnosed with hyperthyroidism and hypercalcemia. Propylthiouracil was then prescribed at 100 mg 3 times a day to correct the hyperthyroidism.

One week later, the patient was admitted to our hospital due to recurrent and exacerbation of symptoms. Physical examination revealed a body temperature of 36.8°C, blood pressure of 116/96 mm Hg, and a heart rate of 98 beats/min. Her body mass index (BMI) was 19.6 kg/m^2^. She was also found to have warm skin, a hand tremor and a moderately enlarged, soft and nontender palpable thyroid gland. Upon admission, the laboratory tests revealed that the FT3 was 10.62 pmol/L, FT4 32.51 pmol/L, TSH 0.014 μIU/mL, antithyroid globulin antibody 18.53 IU/mL (TGAb, normal range: 0–115 IU/mL), thyroglobulin 458.6 μg/mL (TG, normal range: 1.4–78 μg/mL), antithyroid peroxidase antibody 17.06 IU/mL (TPOAb, normal range: 0–34 IU/mL), and TSH receptor antibody 37.01 IU/L (TRAb, normal range: 0–1.75 IU/L). Serum electrolyte levels revealed significantly elevated serum calcium (3.64 mmol/L; adjusted serum calcium 3.74 mmol/L). However, her serum phosphate level was also slightly increased (1.53 mmol/L, normal range: 0.90–1.34 mmol/L) and serum magnesium was normal (0.98 mmol/L, normal range: 0.67–1.04 mmol/L). Her PTH level was significantly low (7.15 pg/mL; normal range: 15–65 pg/mL) and 25-hydroxy vitamin D (25-OH-D) was 12.91 ng/mL (normal range: 20–100 ng/mL). Parathyroid hormone related peptide (PTHrP) was 0.58 pmol/L (normal range: 0–1.31 pmol/L). Urinary Bence-Jones protein was negative and serum alkaline phosphatase (ALP) was 72 IU/L (normal range: 50–135 IU/L), bone alkaline phosphatase (BALP) was 30.1 μg/L (normal range: 2.9–22.6 μg/L), and N-terminal propeptide of type I procollagen (PINP) was 102.6 μg/L (normal range: 20.2–76.3 μg/L). A bone resorption marker, serum C-terminal telopeptides of type I collagen (S-CTX) was 0.92 ng/mL (normal range: 0.36–0.63 ng/mL).

**Figure 1:**
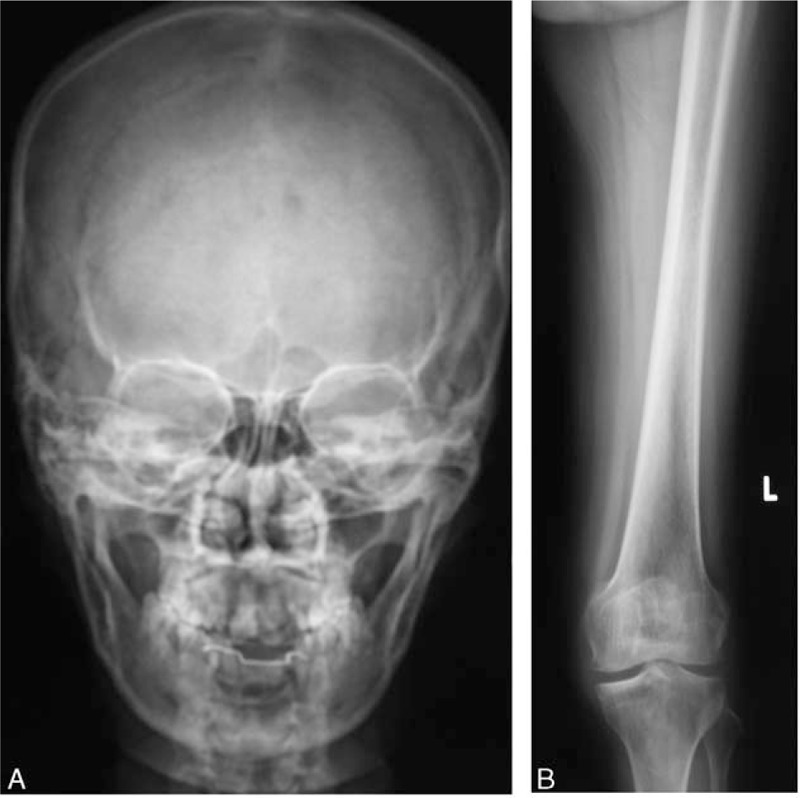
X-ray of skull (A) and left femur (B); X-ray of skull and left femur showed no osteolytic lesions.

**Figure 2:**
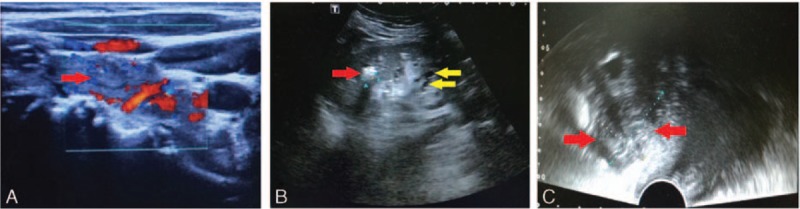
Color ultrasonography examination. (A) Thyroid color ultrasonography showed multiple nodules with high echo (a 21 mm × 11 mm high echo thyroid nodule was represented by a red arrow). (B) Right kidney stone (a single hyperechoic nodule of 11 mm × 8 mm with acoustic shadow, represented by a red arrow), right kidney cysts (represented by yellow arrow); (C) hysteromyoma (red arrow).

Other laboratory findings included serum albumin of 35.0 g/L (normal range: 40.0–55.0 g/L), serum creatinine 140 μmol/L, urea nitrogen 13.21 mmol/L, and uric acid 700 μmol/L. Her electrocardiogram (ECG) showed the sinus rhythm with a slightly prolonged QTc interval (467 milliseconds). Her X-ray examination on chest, thoracic and lumbar vertebrae, left femur and skull were all normal (Fig. 1). Color ultrasonography showed thyroid multiple nodules with high echo patterns (right lobe: 20 mm × 13 mm; left lobe: 10 mm × 8 mm; isthmus: 7 mm × 7 mm) and no malignant lesions were found, hysteromyoma, right kidney stone (a single hyperechoic nodule of 11 mm × 8 mm with acoustic shadow), double kidney cysts (left kidney cysts was no shown), and normal breast and heart structures (Fig. 2). The osteopenia of the lumbar spine (*T* score, L1: −1.3; L2: −1.7; L3: −1.7; L4: −1.3; total lumbar spine: −1.8) and mild osteoporosis of the femoral neck (*T* score: −2.5) were detected by dual-energy X-ray absorptiometry.

**Figure 3:**
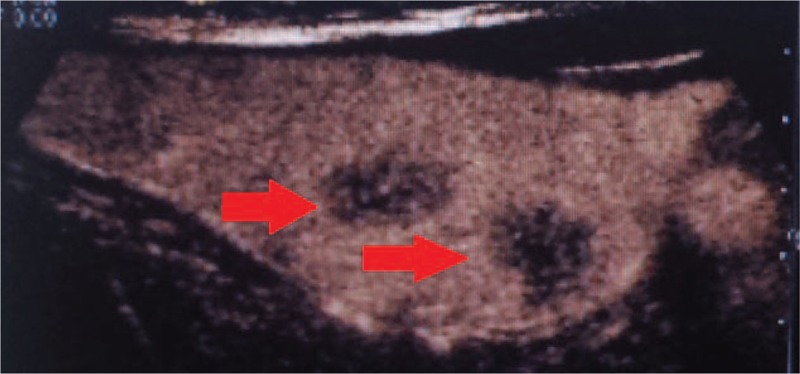
Thyroid ultrasound image: Sulfur hexafluoride contrast-enhanced ultrasound revealed nodular goitre with no malignant changes. Two thyroid nodules (above: 21 mm × 11 mm; below: 14 mm × 21 mm) were represented by red arrows.

In order to clarify the cause of hypercalcemia, we performed further examinations. A second test for parathyroid hormone showed that PTH levels remained low (7.71 pg/mL). Twenty-four-hour urinary electrolytes showed that urine calcium was 9.23 mmol/24 hours (normal range: 2.5–7.5 mmol/24 hours), urine phosphorus 11.99 mmol/24 hours (normal range: 16.10–42.0 mmol/24 hours), urine magnesium 1.63 mmol/24 hours (normal range: 1.00–10.51 mmol/24 hours), and urine sodium 96.0 mmol/24 hours (normal range: 130.0–217.0 mmol/24 hours). To further investigate the existence of any malignant tumor, sulfur hexafluoride contrast-enhanced ultrasound was performed and revealed a nodular goitre (Fig. 3). Her serum IL-6 level was significantly increased (13.7 pg/mL, normal range: 2.2–5.1 pg/mL). Her cortisol level was normal (8 am: 31.1 μg/dL, normal range: 26.5–41.3 μg/dL; 4 pm: 12.1 μg/dL, normal range: 9.2–18.4 μg/dL). Her tumor markers (carbohydrate antigen 125, CA125; carbohydrate antigen 19-9, CA19-9; carbohydrate antigen 242, CA 242; carbohydrate antigen 15-3, CA 15-3; neuron-specific enolase, NSE; human chorionic gonadotropin, HCG; total prostate-specific antigen, tPSA; free prostate-specific antigen, fPSA; alpha fetal protein, AFP; cancer embryo antigen, CEA; growth hormone, GH) were all normal except for a high serum ferritin (494.47 ng/mL; normal range: <219 ng/mL). Whole body Positron emission tomography-computed tomography (PET-CT) scan was then performed and scans showed no abnormalities. Based on the patient's symptoms including palpitations, insomnia, diarrhea, weight loss, and lab results of elevated thyroid hormone level a marked elevated serum calcium level, and lower BMD, a diagnosis of Graves’ disease, hyperthyroidism associated hypercalcemia and osteoporosis was made after further excluding the others causes lead to hypercalcemia.

**Figure 4:**
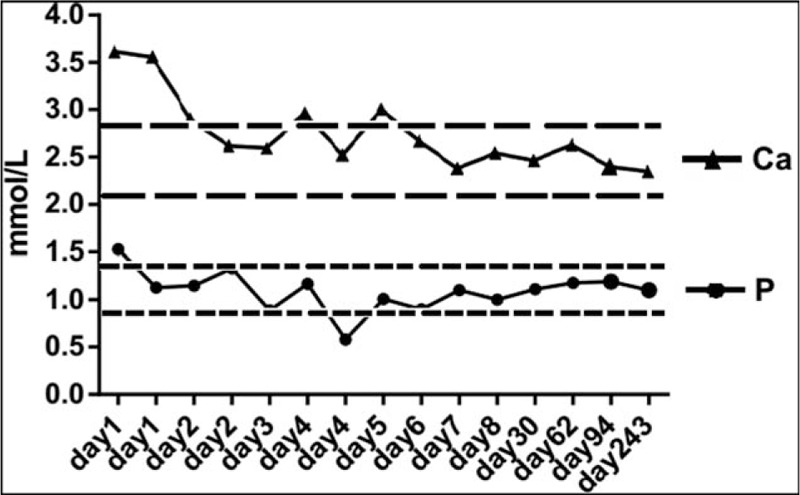
Serum calcium and phosphate levels after treatment of hyperthyroidism and hypercalcemic crisis. Area confined by 2 short dash lines represented the normal range of serum calcium (between 2.08 and 2.80 mmol/L) and area confined by 2 long dash lines represented the normal range of serum phosphate (between 0.90 and 1.34 mmol/L). Ca = calcium, P = phosphate.

Treatment with methimazole 10 mg 3 times daily to correct the hyperthyroidism and propranol 10 mg 3 times daily to control her heart rate and metabolic rate were initiated. Treatment of the patient's hypercalcemia was achieved by intravenous infusion of 6000 mL physiological saline in the first 24 hours, subcutaneous injection of 100 IU salmon calcitonin and intravenous injection of 20 mg furosemide every 12 hours. Her serum calcium level quickly returned to normal after 24 hours of treatment, although it fluctuated at days 4 and 5 (Fig. 4). Three days later, her symptoms of anorexia, nausea, vomit, and malaise were markedly resolved. After 4 days, her renal function returned to baseline (serum creatinine: 99 μmol/L; urea nitrogen 4.98 mmol/L; uric acid 406 μmol/L). The patient was discharged from the hospital with a normal serum calcium level of 2.65 mmol/L after 7 days of treatment. She was asked to continue methimazole treatment for 3 months until thyroid level was stabilized and then was asked to take radioactive iodine therapy.

After 1 month, she was admitted to the local hospital for a checkup. Her serum calcium was 2.55 mmol/L and phosphorus 1.23 mmol/L. Her TSH was 0.03 μIU/mL, FT3 5.07 pmol/L, FT4 12.7 pmol/L, and TRAb 40.01 IU/L. Two months later, both serum calcium (2.71 mmol/L) and phosphate (1.18 mmol/L) were normal. PTH (18.72 pg/mL), 25-OH-D (30.23 ng/mL) and her thyroid function were also found to be normal. Three months later, her calcium (2.52 mmol/L) and phosphorus (1.19 mmol/L) and normal thyroid function were found. Eight months later, her calcium (2.47 mmol/L) and phosphorus (1.10 mmol/L), as well as her thyroid function remained normal (Fig. 4).

## 3 Discussion

Here we presented a rare case of Graves’ disease with associated hypercalcemia crisis in a 58-year-old female. A timely control of calcium level by quick rehydration and antithyroid treatment was critical for her condition.

The most common cause of hypercalcemia is primary hyperparathyroidism.^[10]^ However, in this case, serum PTH levels were decreased and no parathyroid adenoma was found by ultrasonography. Furthermore, osteolytic bone diseases such as multiple myeloma, Paget disease, or bone metastases were also excluded based on normal ALP, PTHrP, cortisol, and urinary Bence-Jones protein. Malignancy related hypercalcemia was further excluded by a negative PET-CT scan. Therefore, the hypercalcemia present in this patient was nonparathyroid hormone mediated. We next excluded medication-related hypercalcemia based on lack of history of medications, in particular, vitamin D. Tumor-related hypercalcemia was also excluded due to negative tumor serum markers, ultrasonography, and PET-CT scans. More importantly, her serum calcium was quickly decreased after rehydration and antithyroid treatment. Therefore, the main cause of hypercalcemia was considered as Graves’ disease-associated hypercalcemia. It is important to note that her symptoms were still exacerbated after treating against hyperthyroidism for 1 week. After ruling out other factors that might trigger the hypercalcemia crisis including infection, medication, or psychological factors, we speculated that this hypercalcemia crisis was triggered by uncontrolled hyperthyroidism and hypercalcemia status. The plasma half-life of T4 is 7 days and the release of thyroid hormones stored in the thyroid gland takes 2 weeks. Therefore, it remained possible that the patient's thyroid hormone level was still high and the uncontrolled hypercalcemia caused hypercalcemic crisis.

Hypercalcemia related kidney stone is common however it usually has multiple lesions. This patient denied previous history of kidney stone diseases and only had one lesion. Although we cannot exclude other causes leading to the right kidney stone including genetic, dietary or life styles causes, we speculated that hyperthyroidism caused hypercalcemia may be one of the important reasons, considering that she had significantly higher calcium excretion level. Besides, we speculated may be calcium sensing receptor (CASR) was abnormal in this patient and no mutation on exon 2–7 was found, We are now doing further sequencing on her whole CASR gene.

The underlying pathophysiology of hyperthyroidism-associated hypercalcemia remains poorly understood. Some studies have explored the mechanisms by which thyroid hormone alters calcium metabolism.^[2,9]^ Increased serum ALP level was found in approximately 50% of the patients with hyperthyroidism complicated with hypercalcemia.^[2,8,11]^ Whereas in our case, in spite of the normal ALP level, the bone formation markers (BALP and PINP), and bone resorption marker were all increased. Mundy et al^[12]^ found that fetal rat long bones cultured with thyroid hormone had significantly elevated osteoclastic activity with an increase in calcium release of 10% to 60%. Previous studies have also suggested that the molecular mechanisms by which a hyperthyroid state effects bone included increased sensitivity of β adrenergic receptors to catecholamines as well as bone to PTH.^[13,14]^ Another clinical study demonstrated that hyperthyroid patients showed increased cortical porosity and resorption compared to healthy controls.^[15]^ Serum IL-6 and its soluble receptor have been shown to positively correlate with thyroid hormone level in hyperthyroid patients. In our patient, serum IL-6 level also significantly increased, which was consistent with previously reported studies.^[16–19]^ Thyroid hormone is also able to directly increase the sensitivity of bones to IL-6, which promotes osteoclastic differentiation via increasing the expression of the receptor activator of nuclear factor κB ligand (RANK-L).^[2,20]^ In addition, some hormones, such as adrenaline and glucocorticoid hormones, are dysregulated under hyperthyroid conditions, thereby contributing to a hypercalcemic state.^[6]^

The primary treatment for hyperthyroidism-associated hypercalcemia is to control the hyperthyroid status. The rapid improvement in her symptoms may due to quick rehydration; however, antithyroid therapy could improve the hyperthyroidism symptoms and maintain the blood calcium level.^[21]^ In this patient with a hypercalcemic crisis, we did not use bisphosphonates drugs and the serum calcium returned to normal levels after 1 day with symptoms resolving 3 days following the treatment. We asked the patient to take radioactive iodine therapy 3 months later. However, because of the fear of hypothyroidism, and the fact that her thyroid level has been stabilized, she was not willing to take this treatment.

Due to elevated serum calcium levels, PTH levels are suppressed and subsequently 1,25-OH_2_D_3_ levels were also decreased. Therefore, calcium from intestinal absorption and renal tubular reabsorption were also decreased. In this patient, PTH was restored to normal range, whereas FT3 and FT4 were normalized after 2 months. Although hypercalcemia often leads to decreased serum phosphate levels,^[22]^ decreased PTH levels may cause increases in the reabsorption of phosphate in the kidney tubules. A few previously published studies, including the findings outlined here, have shown that hypercalcemic patients can have low to normal serum phosphate levels.^[23]^

In conclusion, hyperthyroidism-associated hypercalcemia crisis is a rare complication in hyperthyroid patients; however, this cause should not be ignored after excluding other causes of hypercalcemia. Timely treatment of hypercalcemia is a critical step for rapidly control of symptoms and saving the lives of the patients. Nevertheless, the treatment of hyperthyroidism is beneficial to improve the symptoms and maintain the blood calcium level.

## Acknowledgment

We would like to thank Medjaden Bioscience Limited for their assistance in the preparation of this manuscript.
